# (*E*)-*N*′-(5-Chloro-2-hydroxy­benzyl­idene)-2-nitro­benzohydrazide

**DOI:** 10.1107/S1600536809033108

**Published:** 2009-08-26

**Authors:** Heng-Yu Qian, Da-Ping Qu

**Affiliations:** a Key Laboratory of Surface and Interface Science of Henan, School of Material & Chemical Engineering, Zhengzhou University of Light Industry, Zhengzhou 450002, People’s Republic of China; b Department of Chemistry, Dalian Teacher College, Dalian 116000, People’s Republic of China

## Abstract

In the title Schiff base compound, C_14_H_10_ClN_3_O_4_, the mol­ecule adopts an *E* geometry with respect to the C=N bond and an intra­molecular O—H⋯N hydrogen bond is present. The benzene rings form a dihedral angle of 73.4 (2)°. In the crystal, inversion dimers linked by pairs of N—H⋯O hydrogen bonds occur.

## Related literature

For a related structure and background, see: Qian & Qu (2009 ▶).
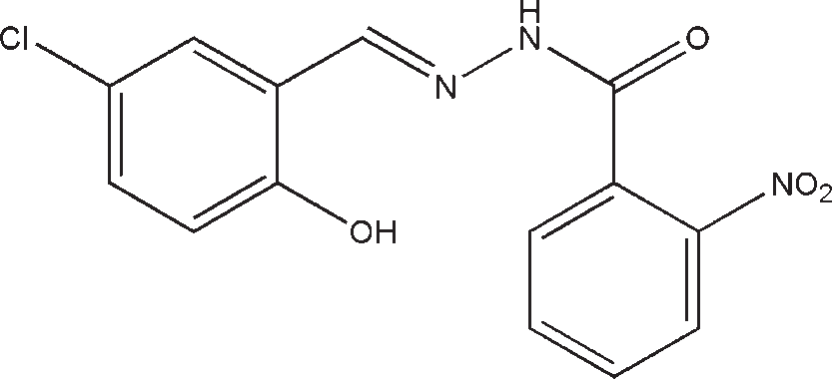


## Experimental

### 

#### Crystal data

C_14_H_10_ClN_3_O_4_
*M* *_r_* = 319.70
Triclinic, 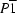

*a* = 7.353 (1) Å
*b* = 10.005 (2) Å
*c* = 10.273 (2) Å
α = 93.393 (3)°
β = 108.144 (3)°
γ = 98.886 (4)°
*V* = 704.9 (2) Å^3^
*Z* = 2
Mo *K*α radiation
μ = 0.29 mm^−1^
*T* = 298 K
0.23 × 0.20 × 0.20 mm

#### Data collection

Bruker SMART CCD diffractometer
Absorption correction: multi-scan (*SADABS*; Bruker, 2001 ▶) *T*_min_ = 0.936, *T*_max_ = 0.944
4324 measured reflections
3004 independent reflections
2237 reflections with *I* > 2σ(*I*)
*R*_int_ = 0.013

#### Refinement

*R*[*F* ^2^ > 2σ(*F* ^2^)] = 0.046
*wR*(*F* ^2^) = 0.122
*S* = 1.02
3004 reflections
203 parameters
1 restraint
H atoms treated by a mixture of independent and constrained refinement
Δρ_max_ = 0.30 e Å^−3^
Δρ_min_ = −0.44 e Å^−3^

### 

Data collection: *SMART* (Bruker, 2007 ▶); cell refinement: *SAINT* (Bruker, 2007 ▶); data reduction: *SAINT*; program(s) used to solve structure: *SHELXTL* (Sheldrick, 2008 ▶); program(s) used to refine structure: *SHELXTL*; molecular graphics: *SHELXTL*; software used to prepare material for publication: *SHELXTL*.

## Supplementary Material

Crystal structure: contains datablocks global, I. DOI: 10.1107/S1600536809033108/hb5055sup1.cif

Structure factors: contains datablocks I. DOI: 10.1107/S1600536809033108/hb5055Isup2.hkl

Additional supplementary materials:  crystallographic information; 3D view; checkCIF report

## Figures and Tables

**Table 1:** Hydrogen-bond geometry (Å, °)

*D*—H⋯*A*	*D*—H	H⋯*A*	*D*⋯*A*	*D*—H⋯*A*
O1—H1⋯N1	0.82	1.94	2.657 (2)	145
N2—H2⋯O2^i^	0.902 (10)	1.966 (11)	2.863 (2)	173 (3)

Symmetry code: (i) 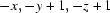.

**Fig. 1.**
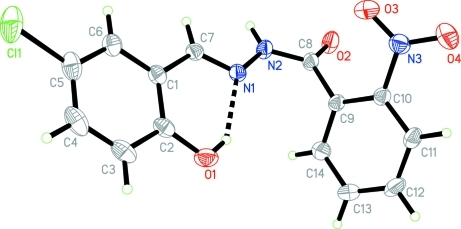
The molecular structure of (I). Displacement ellipsoids are drawn at the 30% probability level. Hydrogen bonding is shown by dashed lines.

**Fig. 2.**
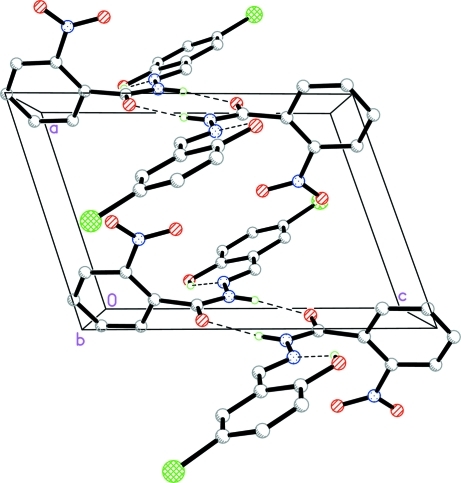
The molecular packing of (I), viewed along the b axis. Hydrogen bonding is shown in dashed lines.

## supplementary crystallographic information

### Comment

As part of our ongoing studies of Schiff bases (Qian & Qu, 2009), we now report the synthsis and structure of the title compound, (I), (Fig. 1).

In the title compound, the Schiff base molecule adopts an *E* geometry with respect to the CN bond, as shown in Fig. 1. There forms an intramolecular O—H···N hydrogen bond. The two benzene rings forms a dihedral angle of 73.4 (2)°. The dihedral angle between the O3/N3/O4 plane and the C9—C14 benzene ring is 23.2 (2)°. In the crystal structure, the adjacent two Schiff base molecules are linked through intermolecular N—H···O hydrogen bonds (Table 1) to form a dimer (Fig. 2).

### Experimental

2-Nitrobenzohydrazide (1 mmol, 0.181 g) and 5-chlorosalicylaldehyde (1 mmol, 0.156 g) were dissolved in anhydrous methanol (15 ml). The mixture was stirred for several minutes at room temperature. The product was isolated and recrystallized from methanol, colorless blocks of (I) were obtained after a week.

### Refinement

The imino H atom was located in a difference map and its positional parameters were refined with a fixed isotropic thermal parameter of 0.08 Å^2^. Other H atoms were positioned geometrically and refined as riding with C—H = 0.93 Å, O—H = 0.82 Å, and with *U*_iso_(H) = 1.2*U*_eq_(C) and 1.5*U*_eq_(O).

### Crystal data

**Table:**

C_14_H_10_ClN_3_O_4_	*Z* = 2
*M**_r_* = 319.70	*F*(000) = 328
Triclinic, *P*1	*D*_x_ = 1.506 Mg m^−^^3^
Hall symbol: -P 1	Mo *K*α radiation, λ = 0.71073 Å
*a* = 7.353 (1) Å	Cell parameters from 1356 reflections
*b* = 10.005 (2) Å	θ = 2.4–25.6°
*c* = 10.273 (2) Å	µ = 0.29 mm^−^^1^
α = 93.393 (3)°	*T* = 298 K
β = 108.144 (3)°	Block, colorless
γ = 98.886 (4)°	0.23 × 0.20 × 0.20 mm
*V* = 704.9 (2) Å^3^	

### Data collection

**Table:**

Bruker SMART CCD diffractometer	3004 independent reflections
Radiation source: fine-focus sealed tube	2237 reflections with *I* > 2σ(*I*)
graphite	*R*_int_ = 0.013
ω scans	θ_max_ = 27.0°, θ_min_ = 2.1°
Absorption correction: multi-scan (*SADABS*; Bruker, 2001)	*h* = −9→9
*T*_min_ = 0.936, *T*_max_ = 0.944	*k* = −9→12
4324 measured reflections	*l* = −13→12

### Refinement

**Table:**

Refinement on *F*^2^	Primary atom site location: structure-invariant direct methods
Least-squares matrix: full	Secondary atom site location: difference Fourier map
*R*[*F*^2^ > 2σ(*F*^2^)] = 0.046	Hydrogen site location: inferred from neighbouring sites
*wR*(*F*^2^) = 0.122	H atoms treated by a mixture of independent and constrained refinement
*S* = 1.02	*w* = 1/[σ^2^(*F*_o_^2^) + (0.0501*P*)^2^ + 0.3008*P*] where *P* = (*F*_o_^2^ + 2*F*_c_^2^)/3
3004 reflections	(Δ/σ)_max_ < 0.001
203 parameters	Δρ_max_ = 0.30 e Å^−^^3^
1 restraint	Δρ_min_ = −0.44 e Å^−^^3^

### Special details

**Table:**

Geometry. All e.s.d.'s (except the e.s.d. in the dihedral angle between two l.s. planes) are estimated using the full covariance matrix. The cell e.s.d.'s are taken into account individually in the estimation of e.s.d.'s in distances, angles and torsion angles; correlations between e.s.d.'s in cell parameters are only used when they are defined by crystal symmetry. An approximate (isotropic) treatment of cell e.s.d.'s is used for estimating e.s.d.'s involving l.s. planes.
Refinement. Refinement of *F*^2^ against ALL reflections. The weighted *R*-factor *wR* and goodness of fit *S* are based on *F*^2^, conventional *R*-factors *R* are based on *F*, with *F* set to zero for negative *F*^2^. The threshold expression of *F*^2^ > σ(*F*^2^) is used only for calculating *R*-factors(gt) *etc*. and is not relevant to the choice of reflections for refinement. *R*-factors based on *F*^2^ are statistically about twice as large as those based on *F*, and *R*- factors based on ALL data will be even larger.

### Fractional atomic coordinates and isotropic or equivalent isotropic displacement parameters (Å^2^)

**Table:**

	*x*	*y*	*z*	*U*_iso_*/*U*_eq_	
Cl1	0.54671 (11)	−0.14057 (9)	0.86494 (8)	0.0837 (3)	
N1	0.1470 (2)	0.23302 (15)	0.43169 (16)	0.0372 (4)	
N2	0.0876 (3)	0.35676 (17)	0.43461 (17)	0.0422 (4)	
N3	0.3643 (3)	0.53956 (19)	0.2180 (2)	0.0510 (5)	
O1	0.1405 (2)	−0.01201 (16)	0.31361 (15)	0.0534 (4)	
H1	0.1202	0.0663	0.3150	0.080*	
O2	−0.0068 (3)	0.53476 (15)	0.32927 (15)	0.0574 (5)	
O3	0.4103 (3)	0.54714 (19)	0.34314 (18)	0.0735 (5)	
O4	0.4446 (3)	0.6178 (2)	0.1575 (2)	0.0898 (7)	
C1	0.2638 (3)	0.05581 (19)	0.5597 (2)	0.0383 (4)	
C2	0.2290 (3)	−0.0378 (2)	0.4431 (2)	0.0428 (5)	
C3	0.2854 (3)	−0.1637 (2)	0.4603 (3)	0.0543 (6)	
H3	0.2585	−0.2270	0.3837	0.065*	
C4	0.3798 (4)	−0.1956 (2)	0.5886 (3)	0.0615 (7)	
H4	0.4185	−0.2796	0.5990	0.074*	
C5	0.4176 (3)	−0.1023 (3)	0.7029 (3)	0.0551 (6)	
C6	0.3583 (3)	0.0212 (2)	0.6893 (2)	0.0463 (5)	
H6	0.3816	0.0820	0.7673	0.056*	
C7	0.2062 (3)	0.18811 (19)	0.5484 (2)	0.0377 (4)	
H7	0.2125	0.2412	0.6277	0.045*	
C8	0.0525 (3)	0.4269 (2)	0.3254 (2)	0.0404 (5)	
C9	0.0740 (3)	0.36796 (19)	0.19420 (19)	0.0369 (4)	
C10	0.2071 (3)	0.43017 (19)	0.13654 (19)	0.0374 (4)	
C11	0.2035 (3)	0.3885 (2)	0.0051 (2)	0.0461 (5)	
H11	0.2919	0.4344	−0.0323	0.055*	
C12	0.0674 (4)	0.2784 (2)	−0.0695 (2)	0.0529 (6)	
H12	0.0638	0.2488	−0.1580	0.063*	
C13	−0.0624 (4)	0.2123 (2)	−0.0142 (2)	0.0573 (6)	
H13	−0.1526	0.1366	−0.0643	0.069*	
C14	−0.0606 (4)	0.2574 (2)	0.1164 (2)	0.0516 (6)	
H14	−0.1516	0.2126	0.1522	0.062*	
H2	0.072 (4)	0.392 (3)	0.5126 (18)	0.080*	

### Atomic displacement parameters (Å^2^)

**Table:**

	*U*^11^	*U*^22^	*U*^33^	*U*^12^	*U*^13^	*U*^23^
Cl1	0.0709 (5)	0.0970 (6)	0.0952 (6)	0.0360 (4)	0.0260 (4)	0.0547 (5)
N1	0.0494 (10)	0.0296 (8)	0.0381 (9)	0.0096 (7)	0.0212 (8)	0.0020 (7)
N2	0.0654 (11)	0.0356 (9)	0.0368 (9)	0.0178 (8)	0.0281 (8)	0.0055 (7)
N3	0.0529 (11)	0.0467 (11)	0.0539 (12)	0.0027 (9)	0.0224 (9)	−0.0015 (9)
O1	0.0639 (10)	0.0449 (9)	0.0481 (9)	0.0110 (8)	0.0156 (8)	−0.0083 (7)
O2	0.1017 (13)	0.0428 (9)	0.0476 (9)	0.0330 (9)	0.0414 (9)	0.0136 (7)
O3	0.0818 (13)	0.0720 (12)	0.0495 (10)	−0.0114 (10)	0.0125 (9)	−0.0109 (9)
O4	0.0946 (15)	0.0820 (14)	0.0882 (14)	−0.0283 (12)	0.0452 (12)	0.0039 (11)
C1	0.0381 (10)	0.0339 (10)	0.0461 (11)	0.0053 (8)	0.0189 (9)	0.0053 (8)
C2	0.0377 (11)	0.0351 (10)	0.0571 (13)	0.0023 (8)	0.0205 (10)	−0.0005 (9)
C3	0.0524 (13)	0.0341 (11)	0.0804 (17)	0.0077 (10)	0.0291 (13)	−0.0022 (11)
C4	0.0554 (14)	0.0390 (12)	0.103 (2)	0.0170 (11)	0.0389 (15)	0.0186 (13)
C5	0.0443 (12)	0.0553 (14)	0.0752 (16)	0.0167 (10)	0.0253 (12)	0.0291 (12)
C6	0.0459 (12)	0.0451 (12)	0.0527 (12)	0.0102 (9)	0.0207 (10)	0.0112 (10)
C7	0.0441 (11)	0.0336 (10)	0.0386 (10)	0.0053 (8)	0.0194 (9)	0.0001 (8)
C8	0.0553 (12)	0.0350 (10)	0.0369 (10)	0.0098 (9)	0.0227 (9)	0.0046 (8)
C9	0.0488 (11)	0.0337 (10)	0.0322 (9)	0.0115 (8)	0.0168 (8)	0.0035 (8)
C10	0.0444 (11)	0.0355 (10)	0.0349 (10)	0.0108 (8)	0.0150 (9)	0.0034 (8)
C11	0.0558 (13)	0.0521 (13)	0.0388 (11)	0.0154 (10)	0.0242 (10)	0.0080 (9)
C12	0.0706 (16)	0.0597 (14)	0.0300 (10)	0.0190 (12)	0.0163 (10)	−0.0004 (10)
C13	0.0663 (15)	0.0525 (14)	0.0424 (12)	−0.0003 (11)	0.0103 (11)	−0.0083 (10)
C14	0.0580 (14)	0.0488 (13)	0.0476 (12)	−0.0015 (10)	0.0229 (11)	−0.0004 (10)

### Geometric parameters (Å, °)

**Table:**

Cl1—C5	1.738 (3)	C4—C5	1.383 (4)
N1—C7	1.276 (2)	C4—H4	0.9300
N1—N2	1.377 (2)	C5—C6	1.373 (3)
N2—C8	1.336 (2)	C6—H6	0.9300
N2—H2	0.902 (10)	C7—H7	0.9300
N3—O3	1.217 (2)	C8—C9	1.503 (2)
N3—O4	1.221 (2)	C9—C14	1.380 (3)
N3—C10	1.460 (3)	C9—C10	1.383 (3)
O1—C2	1.347 (3)	C10—C11	1.380 (3)
O1—H1	0.8200	C11—C12	1.373 (3)
O2—C8	1.228 (2)	C11—H11	0.9300
C1—C6	1.389 (3)	C12—C13	1.364 (3)
C1—C2	1.407 (3)	C12—H12	0.9300
C1—C7	1.451 (3)	C13—C14	1.385 (3)
C2—C3	1.390 (3)	C13—H13	0.9300
C3—C4	1.367 (4)	C14—H14	0.9300
C3—H3	0.9300		
			
C7—N1—N2	115.34 (15)	C1—C6—H6	119.8
C8—N2—N1	122.02 (15)	N1—C7—C1	121.21 (17)
C8—N2—H2	118.8 (18)	N1—C7—H7	119.4
N1—N2—H2	119.2 (18)	C1—C7—H7	119.4
O3—N3—O4	123.3 (2)	O2—C8—N2	120.66 (17)
O3—N3—C10	118.26 (18)	O2—C8—C9	120.00 (17)
O4—N3—C10	118.47 (19)	N2—C8—C9	119.22 (17)
C2—O1—H1	109.5	C14—C9—C10	117.09 (18)
C6—C1—C2	118.97 (19)	C14—C9—C8	119.38 (18)
C6—C1—C7	119.03 (18)	C10—C9—C8	122.92 (18)
C2—C1—C7	121.99 (18)	C11—C10—C9	122.36 (19)
O1—C2—C3	117.59 (19)	C11—C10—N3	117.72 (18)
O1—C2—C1	122.93 (18)	C9—C10—N3	119.85 (17)
C3—C2—C1	119.5 (2)	C12—C11—C10	119.0 (2)
C4—C3—C2	120.7 (2)	C12—C11—H11	120.5
C4—C3—H3	119.6	C10—C11—H11	120.5
C2—C3—H3	119.6	C13—C12—C11	120.11 (19)
C3—C4—C5	119.7 (2)	C13—C12—H12	119.9
C3—C4—H4	120.1	C11—C12—H12	119.9
C5—C4—H4	120.1	C12—C13—C14	120.3 (2)
C6—C5—C4	120.7 (2)	C12—C13—H13	119.9
C6—C5—Cl1	119.7 (2)	C14—C13—H13	119.9
C4—C5—Cl1	119.51 (19)	C9—C14—C13	121.1 (2)
C5—C6—C1	120.3 (2)	C9—C14—H14	119.4
C5—C6—H6	119.8	C13—C14—H14	119.4

### Hydrogen-bond geometry (Å, °)

**Table:**

*D*—H···*A*	*D*—H	H···*A*	*D*···*A*	*D*—H···*A*
O1—H1···N1	0.82	1.94	2.657 (2)	145
N2—H2···O2^i^	0.90 (1)	1.97 (1)	2.863 (2)	173 (3)

Symmetry codes: (i) −*x*, −*y*+1, −*z*+1.
